# Custom Sectional Impression Tray With Sectional Handle for Microstomia Patients

**DOI:** 10.7759/cureus.29433

**Published:** 2022-09-21

**Authors:** Ramesh Kunusoth, Shreya Colvenkar, Suman Thotapalli, Aditya Mohan Alwala, Rathod Prakash

**Affiliations:** 1 Department of Oral and Maxillofacial Surgery, MNR Dental College and Hospital, Sangareddy, IND; 2 Department of Prosthodontics, MNR Dental College and Hospital, Sangareddy, IND; 3 Department of Prosthodontics, SB Patil Dental College and Hospital, Bidar, IND

**Keywords:** impression, handle, impression tray, sectional, microstomia

## Abstract

Microstomia presents a unique challenge to the dentist as well as the patient. Microstomia patients often face difficulty in inserting or removing removable dental prostheses due to constricted opening of the oral cavity. Dentists often face difficulty in inserting impression trays in these patients because of the limited mouth opening. Standard impression procedures need to be modified in such patients. This article describes a simple design for the fabrication of a sectional handle for a definitive impression in microstomia patients. The sectional handle could be sterilized and reused.

## Introduction

Microstomia is defined as a small oral aperture, and it can cause not only functional impairment but cosmetic problems as well. Functional impairments such as problems with speech, food intake, and compromised oral hygiene have been reported in patients with limited mouth opening. Microstomia can be acquired or congenital, the latter being rarely reported. Acquired microstomia can be caused due to burns [1], physical or chemical trauma [2], head and neck radiotherapy [2], injury, surgical treatment of oral cancers [3], or clinical manifestation of systemic diseases like scleroderma.

Various treatment modalities have been described in the literature for the management of microstomia patients, including surgery and microstomia orthoses. Dentists face difficulty during the fabrication of dentures for these patients because of their limitation in mouth opening. The primary hurdle during denture fabrication is the insertion of a standard stock tray and a custom tray. A review of the literature shows various designs for connecting sectional trays [4-10].

This technical report describes a simple technique for designing a cobalt-chrome sectional handle for a custom impression tray. This customized handle can be sterilized and reused.

## Technical report

Handle design

**Figure 1:**
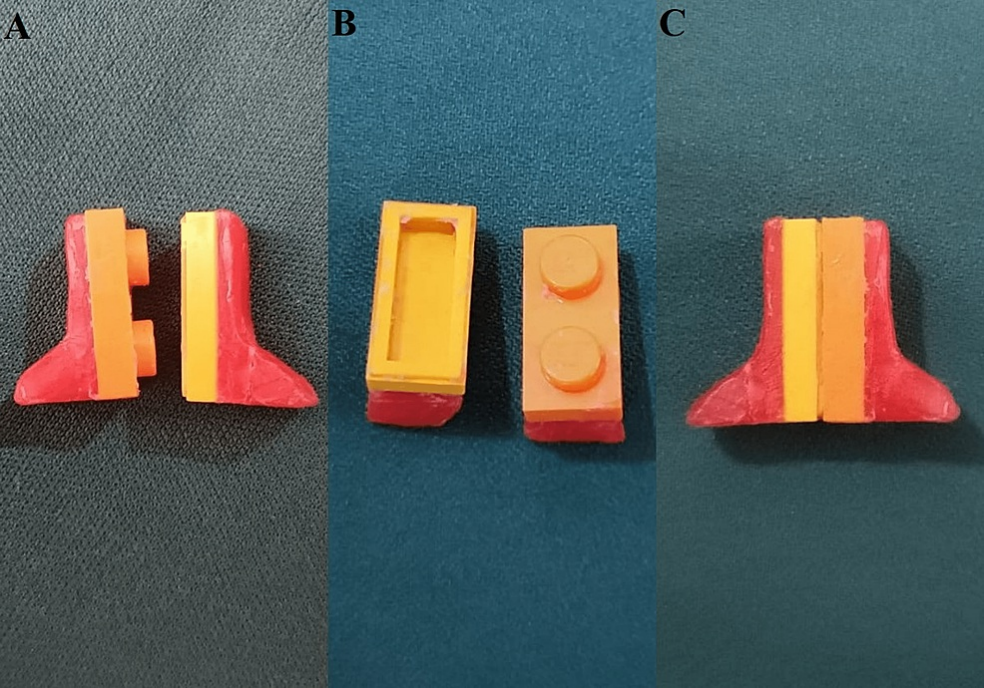
Sectional handle with lego blocks A: male and female segments; B: inner surface; C: interlocked handle

The handle is designed in two parts: it has a male and a female unit. The male and female units are made with wax and lego blocks. Lego blocks are attached to it such that they correctly interlock (Figure 1).

**Figure 2:**
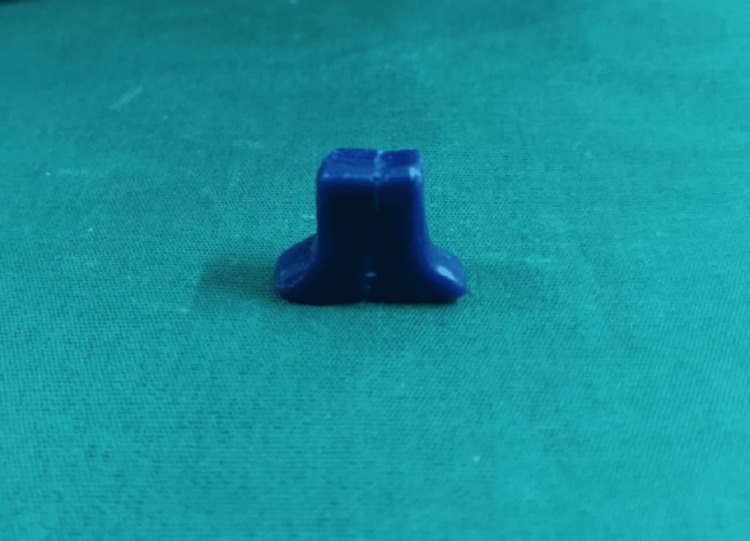
Sectional handle in inlay wax

Both the male and female units are duplicated in inlay wax (Figure 2).

**Figure 3:**
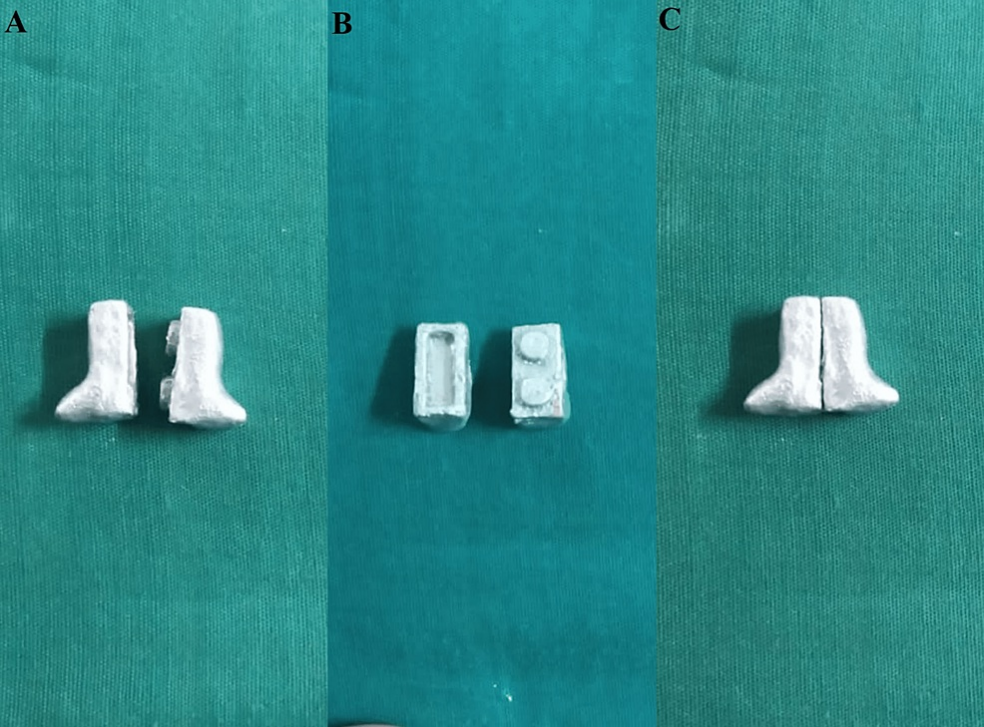
Sectional handle A: male and female segments; B: inner surface; C: interlocked handle

They are then invested and cast in a cobalt-chromium alloy (Figure 3).

Fabrication of custom impression tray and secondary impression

The mandibular tray is fabricated in two sections. Apply separating media (Cold Mold Seal, DPI Pvt. Ltd, Mumbai, India) to the cast and adapt wax spacer over it and section in the midline. Fabricate the right half of the tray with auto-polymerized acrylic resin (DPI RR Cold Cure, DPI Pvt. Ltd) and attach the male unit of the handle in the anterior region of the tray. On completion of setting reaction, apply the separating medium along the midline of the right half of the tray. Fabricate the left half of the tray with auto-polymerized acrylic resin.

**Figure 4:**
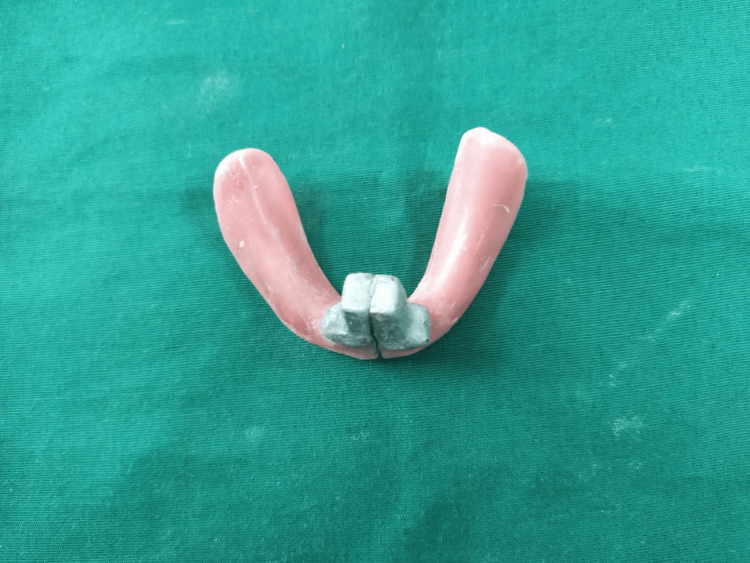
Custom impression tray with interlocked sectional handle

Attach the female unit of the handle to the left half of the tray such that it correctly interlocks with the male unit of the handle (Figure 4).

Carry out the same procedure for the attachment of the handle for the maxillary tray together with the posterior lock. Check the tray intraorally for correct extensions. Carry out border molding for each half of the tray with low-fusing impression compound. Following this, make secondary impressions with zinc oxide eugenol impression paste (DPI impression paste, DPI Pvt. Ltd). Load the impression paste into the right half of the tray and insert it in the patient's mouth followed by the left half of the sectional tray with the impression paste. On setting the impression material, separate sectional trays intraorally and reassemble them externally.

## Discussion

Microstomia presents a challenge to the patient as well as the dentist. Dentists need to make changes in conventional procedures during denture fabrication. Various sectional and collapsible dentures have been described in the literature. Sectional dentures are connected with the help of different attachments that lock the dentures in place [5-10].

The primary hurdle during the fabrication of dentures is making an impression. Limited oral opening causes difficulty in the insertion and removal of the impression tray. The key requirement of the sectional tray is the ease of reassembling and disassembling the tray in the patient’s mouth. Hence, the locking mechanism should be simple and not complicated. The main advantage of this design is that it could be easily reassembled and disassembled in patients' mouths as well as outside. Both elastomeric and non-elastomeric impression materials can be used with this technique.

A literature search has revealed various connecting mechanisms such as hinges [4], snap buttons, locking levers [5], plastic blocks [6,7], orthodontic expansion screws [8], magnet systems [9], and parallel pins [10] for fabricating sectional trays. Snap buttons and parallel pins require precision in securing the trays firmly. Locking levers is technique-sensitive and a little complicated. Magnets tend to lose their magnetism with time and need to be remagnetized.

In this technique, the tray is designed in wax using lego blocks. It is then cast with cobalt-chromium alloy. The main advantage of a sectional handle is that it can be sterilized and reused in the future. It can be easily fabricated with materials readily available in a dental laboratory.

The sectional handle is prefabricated, and hence it is easy to make a sectional tray in a very short time for microstomia patients. The sectional handle is cheap and made from durable material.

## Conclusions

Patients with microstomia presenting for prosthetic rehabilitation pose a challenge to the clinician. Getting an accurate impression is the first and crucial step in denture fabrication. Sectional trays should have a simple locking mechanism. The sectional handle is easy to reassemble and dissemble in the patient’s mouth. This technical report describes an economical, quick, and easy method for the fabrication of a sectional custom tray for microstomia patients. It can be sterilized and reused in the future.
